# Supplementary material for: Mechanistic model of hormonal contraception
Source: PLoS Comput Biol. 2020 Jun 29;16(6):e1007848. doi: 10.1371/journal.pcbi.1007848 (PMC7365466; doi:10.1371/journal.pcbi.1007848)
Supplement: Attachment — Submitted filename: ReviewResponse.pdf [file pcbi.1007848.s001.pdf]

Mechanistic model of hormonal contraception

A. Armean Wright[1], Ghassan Fayad[2], James F. Selgrade[1], Mette S. Olufsen[1]

1 Department of Mathematics and Program in Biomathematics, North Carolina State University, Raleigh, NC, USA
2 Merck & Co., Inc., Kenilworth, NJ, USA

We thank the reviewers for their useful comments and suggestions. We have responded to all comments noting our response in italics. Text changed in the manuscript is included in the review response in red with a reference to the page and line number.

Reviewer 1

General Impressions

- Although the results of the present work appear to qualitatively mimic those of experimental work, I believe that a major shortcoming of the submission is the absence of pharmacokinetic/pharmacodynamic considerations outside of the last paragraph of the manuscript. Given regular dosing (daily or weekly), we would expect the transient time of to contraception to be longer, given the time it would take a standard PK model to approach (an oscillatory) steady state. At the very least, the authors should put more thought into discussing the implications of this based on either preliminary results or by running additional simulations to support their claims.

  Response: In the introduction we have elaborated on the transient time from administration of the contraception to the changes in blood levels and on the fact that this study does not include a pharmacokinetics/dynamics (PKPD) model. The revised text (page 3, line 47) reads:
  The model developed in this study, including ovarian autocrine effects, is used to test hormonal contraceptive treatments via oral administration of ethinyl estradiol and progestin. These treatments are modeled by modifying state variables for blood concentrations of E2 and P4. At this time our model does not include a pharmacokinetic and pharmacodynamic (PKPD) model determining how much or how long it takes for specific amounts of oral contraceptives to produce specific changes in the amount of E2 and P4. Therefore, we administer ethinyl estradiol and progestin as concentrations and assume that contraception is attained if model simulations show a reduction in the LH surge to non-ovulatory levels and/or in P4 levels throughout the cycle.

- The treatment protocol meant to be reproduced consisted of 21 days of daily treatment, whereas the model requires constant dosing for 3 months prior to the start of the simulations, presumably to give the model time to approach a stable limit cycle or steady state.

  Response: The reviewer is correct in noticing that contraceptive drugs were added prior to displaying results. This was done to ensure that the model has reached stable behavior. To emphasize this point we have added the following text to the Methods section (page 13, line 256):

  To analyze model dynamics for each contraceptive treatment, model simulations must have reached stable behavior (cyclic or steady state) ensuring that effects of initial conditions have dissipated. To achieve this, we administered the contraceptive drugs three months prior to analyzing simulation results.

- It is difficult to consider the results of the current work as a quantitatively valid approach to comparison with the treatment data, when the assumed treatment protocol is very different from that of the experiment. I recommend the authors seriously consider the discrepancy and attempt to incorporate a the more realistic treatment prior to comparing to the data. Without this final comparison, the work appears to be purely theoretical in nature. If this is the goal of the manuscript, then this should be very plainly stated.

  Response: We agree with the reviewer that this study is qualitative. But without specific PKPD patient specific data, we find that adding a model of this type would be speculative and take away from the main conclusions explaining that contraception can be achieved at a lower dose for the combined treatment. We have addressed this in the discussion (page 28, line 508). The revised text reads:

  Finally, for each synthetic exogenous hormone, the addition of a model accounting for its pharmacokinetics and its specific activity in relation to the corresponding natural hormone would allow for more detailed representation of different treatments. However, without patient specific data, a study of this type would be speculative and could distract from the main conclusions that contraception can be achieved at a lower dose for the combined treatments.

- The question of variability in contraceptive dosing fs an interesting one, especially where hybrid treatments are concerned. On the one hand, the approach used by the authors–modifying an existing framework to account for additional roles for relevant hormones–is appropriate to study such a problem. However, it seems the description of the approach and explanation of why certain modeling choices were made are lacking in this manuscript.

  Response: To answer this question we have added details to the second paragraph of the Methods section (page 7, line 142) describing contraceptive mechanisms. The revised text reads:

The original models by Clark et al. [14] and Margolskee and Selgrade [15] include progesterone's effect on LH synthesis but do not include progesterone's limiting effect on follicle development via sensitivity to FSH. As a result the original models fail to reproduce contraceptive behavior when administration of exogenous progestins. To capture this effect, our new model introduces a growth limiting factor affecting the early follicle development. Similar to progestins, estrogens act through multiple mechanisms. The primary mechanisms are suppression of LH release by the pituitary [28], included in the models [14,15], and bolstering progesterone's contraceptive effect [1,22], which is not accounted for in the original models. In the model presented here, the latter is included by multiplying progesterone, P4, by an increasing function of estrogen, E2.

In order not to repeat this information, we reformulated the introduction providing a more general statement. The revised text (page 3, line 43) reads:

Specifically, the original menstrual cycle models by Clark et al. [14] and Margolskee and Selgrade [15] do not include ovarian autocrine effects, and therefore they cannot predict the contraceptive response to exogenous administration of progestins.

In addition, we have summarized contraception strategies in the paragraph describing contraceptive mechanisms in the Methods section (page 7, line 158), adding the following text:

In summary, contraception can be achieved either by administering exogenous progestins, estrogen, or a combination of the two. This study uses modeling to illustrate that the combined treatment is advantageous because contraception can be achieved by administrating significantly lower doses of each hormone.

Finally, we have modified the section describing how the contraceptive mechanisms are modeled including sub-paragraphs for each mechanism: progestins (page 14, line 275), estrogen (page 14, line 292), and combination dosing (page 14, line 297).

- Descriptions of the work, beginning with the Modeling Contraception section should be written in a more detailed and clear way, especially as far as modeling assumptions and their justification, and methods in exploring the edose-pdose parameter space are concerned.

  Response: As noted above, the Methods section has been revised adding more details about the modeling assumptions. Regarding exploring the edose-pdose parameter space, we have added a paragraph describing the construction of the Hopf bifurction curve in Fig 9 to the Bifurcation Analysis section (page 24, line 424). The new paragraph reads:

  The curve in Fig 9 displays Hopf bifurcations in the (e_{dose}, p_{dose}) space illustrating the relationship between doses and total contraception. This curve is constructed using the software DDE-BIFTOOL [33], which identifies bifurcations for delay differential equations. We know that if $p_{dose} = 0$ og/mL then the system

attains a steady state at $e_{dose} = 92$ pg/mL, see Fig 7. If e_{dose} is decreased from 92 pg/mL, DDE-BIFTOOL finds the Hopf bifurcation at $e_{dose} \approx 90$ pg/mL. We fix the p_{dose} parameter at small increments between 0 and 1.3 pg/mL and search for Hopf bifurcation with respect to the parameter e_{dose} to generate the curve of Hopf bifurcations in Fig 9. Below the curve are periodic solutions of the model (cyclic behavior) and above the curve are steady state solutions ...

- The organizational structure of the paper works as is, but would benefit from a great deal more detail about the simulation methods–in addition to the assumption previously mentioned–particularly with respect to distinguishing between previously used and new parameters, parameter estimation (if applicable), and the major differences between the approach in the paper compared to that of the experiment.

  Response: We have added two new sections describing the data (in the method section after the description of the normal menstrual cycle, page 8, line 162) and summarizing the model (at the end of the Methods section, page 15, line 309).

  The first section describes the data from the normal menstrual cycle extracted from the study by Welt et al. [25], as well as data on progestin only dosing extracted from the study by Obruca et al. [16] and Duijkers et al. [17], and the combined dosing extracted from the study by Mulders and Dieben [18].

  The second new section (page 15, line 309) summarizes the model. This section lists the parameters (nominal and optimized) and outlines the simulations conducted in study.

- In addition, the manuscript would benefit from additional editing for typos, grammar, and general flow of the writing. There are also several terms that are used with inconsistent punctuation (hyphenated, etc.) and improper use of open quotation marks, which I assume are due LaTeX's idiosyncrasies.

  Response: We have read through the complete manuscript correcting all grammatical errors and typos.

Specific Major Comments

1. Page 12-13 Eq (5): It is not clear why the choice is made to separate b and c1 as parameters, particularly when they are both multiplied by the FSH variable and scaled by a function of Papp. Why not just define a single parameter equivalent to b+c1, since these would not be independently identifiable anyway?

   Response: Studying the manuscript we realized there was a typo in the first term of Eq (5). We have revised the model correcting this mistake. The new Eq (5) (page 12) states:

$$\frac{d}{dt}RcF = (b + c_1 RcF)\,\frac{FSH}{(1 + P_{app}/Ki_{RcF,P})^{\xi}} - c_2 LH^{\alpha} RcF$$

2. Eq (16): There appear to be two primary effects of exogenous progestin on the system: (1) the neuroendocrine effects involved in regulating LH synthesis and (2) the ovarian autocrine effect of reducing FSH sensitivity of follicles. The authors do not provide sufficient justification for making the modeling choices reflected in Eq (16). In particular, if E2 is meant to upregulate P4 receptors, effectively increasing P4 function, there is no differentiation between neuroendocrine and ovarian mechanisms. That is, the choice of E2-mediated feedback in Eq (16) seems to reflect how E2 behaves in the hypothalamic-pituitary axis rather than in ovarian tissues (looking at the forms used in the original model). If the feedback modeled is universal in all tissues, then this should be stated explicitly in the manuscript. In addition, if E2 primes P4 receptors for the luteal stage, why would this effect be reflected in follicle growth in the same way that P4 works to inhibit gonadotropins when elevated throughout the luteal stage? Is there a good explanation as to why E2-dependent Papp might affect different tissues from regularly circulating P4? I suggest that the authors explore this possibility for the sake of completeness.

   Response: These are excellent points and we agree with the reviewer. Ideally these effects should be separated, but doing so would add more parameters, which have to be estimated. A task that is difficult do do without additional data, in particular neuroendocrine data. Therefore, we chose a universal approach using just one term describing P4's effect on all tissues. To clarify this we have added the following to the end of the Ovarian Model section (page 12, line 239):

   Eq (16) defines the variable P_{app}, which groups the effects of progesterone on both the brain and the ovaries, i.e., we assume that P4 affects all tissues in the same way. In the ovaries, during follicle development, P4 limits sensitivity to FSH [20,21] as described by the denominator of Eq (5).

   The second term in Eq (5) represents the increase in P4 receptor expression due to E2 [22,23] in the form of an increasing Hill function. Hence, the presence of estrogen enhances the effectiveness of P4.

3. It is also not clear why the priming effect of E2 can only maximally double the effective P4 concentration. Since explicit receptor expression and binding kinetics are not modeled here, it is unclear how the model chosen is mechanistically appropriate. The most general model for Eq (16) might have the form

   $$Papp = (P4/(1 + Z)) * (1 + Z * Hill(E_2)),$$

   where Z is not necessarily 1 (as is currently the case). Have the authors already explored this possibility? If so, this should be mentioned in the manuscript, with a justification of the final form for Eq (16). If not, it may be useful to explore the dynamics of the model for various scalings.

Response: At this stage we have kept the formulation used in the first submission suggesting that the maximum of Papp is P4. This formulation produced the desired effect without adding parameters, but could be modified further with the intent of developing a more general formulation. We have noted this limitation in the discussion (page 28, line 495) adding the following text:

A limitation of the current model is the assumption that the effect of estrogen on progesterone can be combined into one term P_{app} that does not differentiate the neuroendocrine versus the ovarian systems, and that has a maximum of P4. In reality the effect likely differs between organs, and it may be that for large concentrations of E2 the P_{app} is larger than P4, but without additional data we chose this unifying approach. Future studies should explore these possibilities in more detail.

Specific Minor Comments

1. Page 3, Table 1: Are there references for the parameters used, especially those that are part of the modification? Or if most parameters used come from existing models, please also mention this.

   Response: We have added references to the table indicating which parameters are taken from the base model by Margolskee and Selgrade [15]. In addition, we have marked new parameters, and parameters that are estimated. As noted earlier, Table 1 is moved to a new section summarizing the model (page 15, line 309).

2. Page 5, line 99: The wording for "not well understood mechanisms" is awkward. Please consider fixing this.

   Response: We have rephrased the sentence adding a reference. The revised sentence (page 5, line 105) reads:

   During ovulation the follicle ruptures and releases the oocyte through complex mechanisms, for details see [1].

3. Page 7, lines 134-135: Are the functions of LH mentioned here also normal functions of endogenous progesterone?

   Response: As noted by the reviewer, the model includes the effect of endogenous progesterone on LH synthesis and release. This model component was adopted from previous studies [14,15]. A biological justification of this effect can be found in the study by Yen [28]. We have added a reference to the manuscript. The revised text (page 7, line 136) reads:

   The primary mechanisms are suppression of LH release by the pituitary [26], included in the models [14,15], and bolstering progesterone's contraceptive effect [1,22], which is not accounted for in the original models

4. Page 9, Fig 2: The hormones listed in the caption and appearing in the blue boxes include P2. Is this supposed to be P4 instead?

Response: We thank the reviewer for noting this error, we have corrected the typo in Fig 2.

5. Page 10, Eqs (3) and (4): There appears to be a typo in the denominator of the FSH release terms $(1 + c_F SH, E * E_2)$, where E_2 should be squared. The units for $c_F SH, E$ appearing in Table 1 are consistent with the Schlosser and Selgrade model [13], but this term is not.

   Response: We thank the reviewer for noting this error, we have corrected the typo in Eqs (3) and (4).

6. Page 11, lines 217 and 218: The description of estrogen and progestin doses being added lacks subject-verb agreement. Please check the specifics.

   Response: We have corrected this grammatical error in the revised manuscript.

7. Page 13, line 243: The FSH-dependent growth term mentioned is not actually "scaled by a factor proportional to applied progestin" as it appears in Eq (5). The terminology should be fixed in this sentence to match what is reflected in the equation in question.

   Response: We agree with the reviewer on this comment, we have revised the text as follows (page 14, line 283):
   Two growth terms proportional to FSH in Eq (5) are divided by a term including the applied progestin.

8. Page 13, lines 263-264: Statement beginning with "Insufficient gonadotropins..." is incomplete (fragment).

   Response: We acknowledge this error and have corrected the sentence (page 14, line 295), which now reads:

   Insufficient gonadotropins from the pituitary prevent a LH surge.

9. Page 14, lines 275-276: Were any parameters estimated in the model fitting process? If so these should be explicitly indicated, either in the table of parameters or in the Results section. If not, the authors should give a description of how new parameters for the model addition were chosen.

   Response: We have added a new section (page 15, line 309) summarizing the model. This section discusses parameter values. It states which parameters are taking from previous studies and which parameters are estimated.

10. Page 15, Fig 3 & description: How many cycles of data were used in the model fitting? What is the cycle length from the new model without contraceptives compared to the data? Is there any insight to the discrepancy between the model and the data? How does the baseline model (no estrogen/progestin) compare to the original model without the new modifications? An illustration of this might be useful for the average reader.

Response: The 28 day data were repeated three times and parameters were estimated over a time period of 84 days. The Modeling contraception section on page 13, line 261 discusses why new terms must be added to the old model in Margolskee and Selgrade [15] in order to predict contraception;

The new model studied here is based on the model in Margolskee and Selgrade [15] which cannot predict contraceptive behavior. For instance, if a progestin dose of 1.3 ng/mL is administered to the Margolskee and Selgrade model then this dose results in a slightly higher LH profile than the normal. This occurs because a small additional amount of P4 is more effective at increasing FSH production (see Eq (4)) than inhibiting LH production (see Eq (1)). More FSH causes increased early follicular growth (see Eq (5) without the P_{app} term) resulting in more early follicular E2 and hence a slightly higher LH surge. Including the P_{app} term without the E2 enhancement dampens this growth and decreases the LH surge but it still is at an ovulatory level. Thus in order to model progestin's contraceptive effect both the inhibition of P4 on early follicular development and the enhancement due to E2 are needed to predict contraception in this model setting.

11. Page 21, Fig 9: "Low Dose Combination" is spelled incorrectly. Please add units to the axes in the graph as well. Also, it is not at all described how the bifurcation diagram was generated. Was this through ad hoc methods or using some other software? Please clarify. Further, because the manuscript places emphasis on the distinction between total contraception and "partial" contraception with periodic solutions, it would be useful to overlay regions within the periodic solutions for which some level of contraception is obtained (clinical criterion), even if this is not valid mathematically. This also seems relevant in the context of limiting contraception doses to clinically effective values, rather than mathematically effective values. More emphasis on the physiological and clinical relevance would be helpful in the discussion of model results.

    Response: We have corrected the typo on Fig 9 and added units to the axis. In addition, we have added a paragraph in the Bifurcation Analysis section describing how the bifurcation diagram was generated, but to keep the graph simple we have not added the overlay as there is no exact limit separating the regions. As noted earlier, we revised the Bifurcation Analysis section (page 24, line 416).

    In addition, we have added a more detailed discussion of the partial vs. complete contraception in the discussion of model results (page 28, line 485). The revised text reads:

    In clinical settings, the contraceptive effect is reached well before the "total contraception" described by the model. Therefore the modeling results cannot directly be translated to clinical applications, but if combined with a PKPD model, the ideas put forward here has potential to be used in designing new treatment strategies.

12. Page 22, lines 343-344: Please provide references for the half-lives of progestin and estrogen. Also, are the half-lives of exogenously administered hormones different from endogenous ones?

    Response: The elimination half-life values are approximate, they depend on the dosing method, to be more clear we have added references in the revised manuscript. The revised text (page 26, line 449) reads:

    Both elimination half-lives of the drugs are short compared to the model time scale: progestin has an approximate half-life of a day [34,35] and estrogen of two days [36] depending on the dosing method.

13. Page 24, line 388: The authors comment on the shortcoming of the model, which may require that FSH synthesis be modeled using additional reproductive hormones. I assume the authors are referring to inhibin B, among others. If this is the case, the known hormones should be stated explicitly.

    Response: The reviewer is correct, that inhibin B should be added, we have made a note of this in the discussion, see revised text (page 28, line 505).
    To improve the model, Eq (3) needs to be modified so that FSH synthesis depends on more reproductive hormones especially inhibin B.

14. General: In the Results section, it is left unclear whether the doses used in the simulations are used to match the data in reference [15] or whether they reflect the minimum doses required to elicit total (or partial) contraception. The bifurcation diagram certainly matches these values, but it is unclear whether this is a by-product of the model itself or whether the parameters were tuned to match experimental data. Please clarify.

    Response: This is a good point, the dosing reflects the minimum dose needed to obtain contraception. The data used for comparison list dose in mg, yet the model requires a dose in ng/mL (concentrations). For this study we scaled the values used by Obruca et al. [16] and subsequently adjusted them using the bifurcation analysis. To make this clear, we have added the following paragraph to the Progestin Based Contraception section (page 19, line 388):
    The doses giving the hormone levels discussed above from Obruca et al. [16] are in mg, whereas in the model they are given in concentrations. Approximate concentration doses were obtained by converting the high dose values given in the study by Obruca. These were subsequently adjusted to obtain a high dose, representing the lowest concentration giving a constant long-term solution. The low does was set to approximately half the high dose. More specifically, for the high dose $p_{dose} = 1.3$ ng/mL and for the low dose $p_{dose} = 0.6$ ng/mL.

Reviewer 2

General comments

- The paper describes an augmentation of a previously published model of hormonal regulation of the menstrual cycle, through addition of two autocrine terms, and demonstrates model predictions given low and high dosing of estrogen alone, progesterone alone (nominally as progestin), and a combination of the two. The model has potential utility in designing hormone dosing regimens to achieve birth control, characterized by an acyclic state with low LH throughout, and also to elucidate what mechanism of the female reproductive cycle are key to explaining observed responses to birth control regimens. Unfortunately, the paper as is lacks sufficient detail to achieve either of these, but I believe that a small amount of additional work could help it to meet these objectives.

  I also suggest ways in which the results could be significantly improved, but those could be discussed as potential future research. For context, I have experience in the mathematical modeling of endocrine/hormonal systems, but not in contraception science. One way in which the model could be useful is that researchers seeking to refine birth control methods (combinations of estrogen and progesterone capable of achieving birth control) might use it to identify regimens that will achieve this purpose. But to be convinced that it can be used for this purpose, I would want to know that it can accurately predict the result of existing regimens.

  Response: The goal of this paper was to develop a model that can explain dynamic behavior in response to contraceptive treatments highlighting the difference between estrogen based and progestin based protocols. To emphasize the general effects, we did not include a pharmacokinetics/dynamics (PKPD), required to address the questions raised above. Moreover, since we do not have specific PKPD data, we find that adding such component is beyond the scope of this study. To make this point clear to the reader we have added the following text to the introduction (page 3, line 47):

  The model developed in this study, including ovarian autocrine effects, is used to test hormonal contraceptive treatments via oral administration of ethinyl estradiol and progestin. These treatments are modeled by modifying state variables for blood concentrations of E2 and P4. At this time our model does not include a pharmacodynamic model to determine how much or how long it takes for specific amounts of oral contraceptives to produce specific changes in the amount of E2 and P4. Therefore, we administer ethinyl estradiol and progestin as concentrations and assume that contraception is attained if model simulations show a reduction in the LH surge to non-ovulatory levels and/or in P4 levels throughout the cycle.

- The authors demonstrate an ability to simulate partial and complete birth control, but how do the doses used in the modeling that achieve these effects (low and

high estrogen, for example) compare to corresponding clinical doses? There is a brief reference to progestin levels in [15], made towards the end of the introduction, but the specific levels used in those experiments and the current simulations (and rationale for them) should be described in the Methods section on modeling contraception.

Response: We recognize that the description of dosing was not described clearly. For progestin, the dose used in simulations are related to the study by Obruca et al. [16]. We have added a paragraph explaining how the chosen doses relate to values found in this study (see revised text below). For estrogen, no data are available (as mentioned in the section on estrogen-based contraception. More specific details on doses will be difficult to simulate without the inclusion of specific PKPD model as addressed above. The revised section (page 19, line 388) describing the progestin dose reads:

The doses giving the hormone levels discussed above from Obruca et al. [16] are in mg, whereas in the model they are given in concentrations. Approximate concentration doses were obtained by converting their high dose values. These were subsequently adjusted to obtain a high dose, representing the lowest concentration giving a constant long-term solution. The low does was set to approximately half the high dose. More specifically, for the high dose $p_{dose} = 1.3$ ng/mL and for the low dose $p_{dose} = 0.6$ ng/mL.

- Further, the P4 and FSH predictions in Figure 5, for example, are well outside the observed ranges.

  Response: We agree with the reviewer that these levels are outside the observed ranges. In our study P4 includes both the exogenous and endogenous progestin, whereas the study by Obruca et al. [16] data only includes endogenous levels. To make this clear we have added the following to the caption of Fig 5 (page 21).

  For P4 we note a significant difference between model predictions and the data. This likely stems from the fact that in the model P4 includes both endogenous and exogenous progestin, while the data only measure the endogenous levels.

  Response: Further, we recognize that FSH is a weak link in our model, and it may not be possible to get better fits without including inhibin B and other factors. While adding more detail likely will improve predictions of FSH, we do not expect that they will change the main conclusions found in this study. We therefore leave this improvement for future studies. We have added some comments in the Results (page 18, line 380) and Discussion (page 28, line 501) sections addressing this point.

  Results: Notice that in Figs. 4–7 the FSH profiles in response to contraceptive treatments are higher than biologically observed [18]. This occurs in our model because FSH synthesis is suppressed only by inhibin A (see Eq (3)). In our model of contraception, ovulation does not occur so the corpus luteum does not develop

and InhA is produced at low levels (see Eq (17)). Hence, a considerable amount of FSH is synthesized and the FSH profile is high. Inhibin B is produced during the follicular phase of the cycle and would provide inhibition of FSH in a contraceptive situation. However, including inhibin B would complicate the present model significantly

Discussion: Another limitation is that the predicted FSH response to hormonal treatment. Eq (3) indicates that FSH synthesis only depends on inhibin A. When a contraceptive state is reached, luteinization does not occur and so inhibin A is diminished. Because inhibin A inhibits FSH synthesis, the model predicts that in a contraceptive state FSH is produced at a high level. However, this is not observed biologically [18]. To improve the model, Eq (3) needs to be modified so that FSH synthesis depends on more reproductive hormones especially inhibin B.

- Off hand these decrease my confidence in the model's ability to predict hormonal response to contraceptive dosing. At a minimum, I suggest that the authors discuss how the simulated estrogen and progesterone (progestin) compare to doses used in the study which they simulate. Better would be to explore what model parameters or features would need to be adjusted such that all predicted hormone levels are within the experimental range from [15,16,17]. A rough sensitivity analysis could provide the later.

  Response: See above discussion on doses.

- A very interesting qualitative result stated in the paper, but not really shown, is that two autocrine terms had to be added to the ovarian model in order for the birth control regimens to be successfully described.

  Response: We thank the reviewer for this comment. We have added a paragraph in the section describing the contraception model describing why both effects are needed. The revised text (Page 13, line 261) reads:

  The new model studied here is based on the model in Margolskee and Selgrade [15], which cannot predict contraceptive behavior. For instance, if a progestin dose of 1.3 ng/mL is administered to the Margolskee and Selgrade model [15] then this dose results in a slightly higher LH profile than the normal. This occurs because a small additional amount of P4 is more effective at increasing FSH production (see Eq (4)) than inhibiting LH production (see Eq (1)). More FSH causes increased early follicular growth (see Eq (5) without the P_{app} term) resulting in more early follicular E2 and hence a slightly higher LH surge. Including the P_{app} term without the E2 enhancement of Eq (16) in Eq (1) dampens this growth and decreases the LH surge but it still is at an ovulatory level. Thus in order to model progestin's contraceptive effect both the inhibition of P4 on early follicular development and the enhancement due to E2 are needed to model contraception.

  But the authors don't show predictions of the original model, without these terms: how badly does the original model fail to predict contraceptive dosing response?

What happens if only one of the autocrine terms is added? How did the authors determine that both were necessary? In short, show me that *both* additional terms are needed for the model to perform successfully, by showing how it fails without them, or with only one of them.

Response: The new model studied here is based on the model in Margolskee and Selgrade [15] but that model fails to predict contraceptive behavior as explained at the end of the Modeling Contraception section. The revised text is given in the previous response.

- Also, please provide references to biological sources which describe these autocrine mechanisms… reference for the effect of progestins on follicle sensitivity to FSH [15, 20] are given in the Contraceptive Mechanisms section, but these should be repeated in the Ovarian Model section, where the model equations are being described. I did not see references for the autocrine effect of estrogen. The supporting literature/data should be cited along with the description of the corresponding equations.

  Response: The discussion and references for E2 increasing the effectiveness of P4 are presented in the combined hormonal contraception subsection of the Methods section. We state that: "In the combined treatment with estrogen and progestin, estrogen serves to bolster progesterone's effect but also inhibits LH release [1]. The presence of estrogen upregulates progesterone receptor expression, which increases P4's effectiveness. This has been shown in ovine and rat uterine cells [1, 32]. A possible secondary effect of the the mid-cycle rise in estrogen is to prime P4 receptors for the luteal phase [32]." As suggested by the reviewer, we have added references to the Ovarian Model Section (page 12).

- Also, how where the forms and the parameters of the autocrine terms selected? They are fairly standard Hill-like forms, but how were the values of the coefficients obtained? It's fine if this was a simple attempt to fit data, but then that should be stated. Where they arrived at by trial-and-error, or formal optimization? Implicit in the estrogen autocrine term (equation 16) is that the effect varies between a maximum of 1 (100%) and 0.5 (50%). How was this range identified? Are there supporting data? Were other ranges from minimal to maximal induction considered?

  Response: We have added a new section summarizing the model, which in detail discuss all parameters, including the new parameters (15, line 309). In addition, we have added more detail in the section discussing the form of Eq (16). As noted by the reviewer, we chose to use a universal approach using just one term describing P4 effects on all tissues. To clarify this we have added the following to the end of the Ovarian Model section (page 12, line 239). The revised text reads:

  Eq (16) defines the variable P_{app}, which groups the effects of progesterone on both the brain and the ovaries, i.e., we assume that P4 affects all tissues in the same

way. In the ovaries, during follicle development, P4 limits sensitivity to FSH [20,21] as described by the denominator of Eq (5).

The second term in Eq (16) represents the increase in P4 receptor expression due to E2 [22,23] in the form of an increasing Hill function.

Response: This formulation produced the desired effect without adding parameters, but it could be studied further with the intent of developing a more general formulation. We have noted this limitation in the discussion (page 28, line 495) adding the following text:

A limitation of the current model is the assumption that the effect of estrogen on progesterone can be combined into one term P_{app} that does not differentiate the neuroendocrine vs. the ovarian systems, and that its maximum is P4. In reality the effect likely differs between organs, and it may be that for large concentrations of E2 the P_{app} is larger than P4, but without additional data we chose this unifying approach. Future studies should explore these possibilities in more detail.

Specific comments

1. While it is nice to augment figures with color, keep in mind that 10% of people (readers) are color blind. Different line types (short dash, long dash, dot-dash, etc.) and/or colors that are distinguishable when printed in grey-scale should be used.

   Response: In figures 1,2, and 9, we augmented the figure caption referring to colors and the placement of relevant components. Figures 3-8 are plotted with different line types. In addition, we have changed the color shading such that they appear distinct when printed in black and white.

2. P. 5, first line: *usually* a single follicle will advance to ovulation. Sometimes it's more than one! (This is said later but should be said here.)

   Response: This observation is correct, we have revised the text to make this more clear. The revised text (page 5, line 100) reads:

   The follicles compete for FSH (and later LH) and one or more follicles will advance to ovulation, if sufficiently stimulated by gonadotropins.

3. The term "applied progesterone" (for equation 16, first introduced at the bottom of p. 11) lacks biological meaning. The specific autocrine effect being described is induction of the progesterone receptor. A separate term, really equation (16) but for P4, should be defined as the scaled progesterone receptor (PR) expression. Since the maximum of this term is 1, it PR expression / maximal PR expression. The degree of PR activation is then defined as the product of P4 and PR expression. This product could also be called progesterone signal.

   Response: As suggested by the reviewer we have added a more precise definition of P_{app} in the section describing the hypothalamus and pituitary model where the

term first appears. But we have kept original notation throughout the manuscript. The revised definition (page 10, line 197) reads:

P_{app}, defined in Eq (16), is as a product of P_4 and scaled progesterone receptor expression. Hence, P_{app} measures the progesterone signal in the system.

4. I checked the cited references for the original model [12, 13] and another by those authors (Clark et al., 2003) and saw that that version of the model better replicated FSH peak, which this version appears to badly under-predict (Figure 3), and the height of the secondary E2 surge. I suspect that when the autocrine terms were added, the original model parameters were not re-tuned to match these features of the normal cycle. When the model structure was changed, the model parameters should have been revised. Is the low FSH peak (relative to the original model) due to the autocrine terms? Also, how is it that the autocrine terms do not extinguish the normal cycle entirely, without exogenous hormones?

   Response: We agree with the reviewer and have refitted the parameters significantly improving the fits as shown in Figure 3. In addition, we have added error-bars for the Welt data making it easier to judge the quality of the fits. Moreover, this study is based on the model by Margolskee and Selgrade [15], which is fit to data from Welt et al., 1999 because they contain inhibin B. The model in Clark et al. [14], was fitted to data from McLachlan et al. [32]. The McLachlan data are less noisy and therefore gave better predictions, but they do not contain inhibin B. This study does not include inhibin B, and therefore could be fitted against either dataset, but since we used parameters reported by Margolske and Selgrade and hope to add inhibin B in future studies we chose to fit the model against the newer data. We have added a note (listed earlier) about this in the revised manuscript (3rd paragraph, page 17).

5. And it would be much better if the experimental data in Fig 3 were shown with confidence bounds (error bars), since that would allow a reader to judge whether areas where the fit is poor are at least within the range of variability.

   Response: As suggested by the reviewer we have added confidence bounds to Fig 3.

6. Fig 6: the legend says that the LH surge has been eliminated, but there is still a small peak occur every 33 days. I would say "significantly suppressed" instead.

   Response: We agree with the reviewer we have modified the caption for Fig 6 (page 22) as follows:

   In this simulation, LH has a small mid-cycle rise, but the large LH surge is significantly suppressed and ovulation does not occur indicating a contraceptive state, yet the hormone levels still vary during the cycle.

7. Fig 9: again, I'd like to know how the P4 and E2 doses used at the three dose levels compare to those actually used.

Response: We have added a more detailed description of the doses used in the model when they were first introduced (see earlier comments). In addition, we have modified the caption for Fig 9 specifying the low- and high doses.
